# Hydroxytyrosol (HT) Analogs Act as Potent Antifungals by Direct Disruption of the Fungal Cell Membrane

**DOI:** 10.3389/fmicb.2018.02624

**Published:** 2018-11-06

**Authors:** George Diallinas, Nausica Rafailidou, Ioanna Kalpaktsi, Aikaterini Christina Komianou, Vivian Tsouvali, Iliana Zantza, Emmanuel Mikros, Alexios Leandros Skaltsounis, Ioannis K. Kostakis

**Affiliations:** ^1^Department of Biology, National and Kapodistrian University of Athens, Athens, Greece; ^2^Department of Pharmacy, National and Kapodistrian University of Athens, Athens, Greece

**Keywords:** fungal pathogens, *Aspergillus nidulans*, plasma membrane, antimicrobial, resistance

## Abstract

Fungal infections constitute an emerging threat and a prevalent health problem due to increasing number of immunocompromised people and pharmacological or other treatments aiming at viral infections, cancer or allergies. Currently used antifungals suffer from inefficiency, toxic side effects and developing drug-resistance. Additionally, over the last two decades no new classes of antifungals have been approved, emphasizing the urgent need for developing a novel generation of antifungals. Here, we investigate the antifungal activity of a series of chemically synthesized Hydroxytyrosol (HT) analogs. HT is one of the major phenolic compounds in olive oil, shown to possess radical-scavenging antioxidant, antiproliferative, proapoptotic and anti-inflammatory activities. No previous report has studied HT analogs as antifungals. We show that specific analogs have broad and strong antifungal activity, significantly stronger than the parent compound HT. Using *Aspergillus nidulans* as an *in vivo* cellular model system, we show that antifungal HT analogs have an unprecedented efficiency in fungal plasma membrane destruction. Importantly, antifungal HT analogs did not show toxicity in a mammalian cell line, whereas no resistance to HT analogs was obtained by standard mutagenesis. Our results open the way for the development of a novel, efficient and safer class of antifungals.

## Introduction

In recent years, systemic fungal infections have emerged as an increasingly prevalent health problem (McCarthy et al., 2017). Infections are rising among immunocompromised patients, including individuals suffering from HIV/AIDS or diabetes mellitus, or following organ transplantations and immunosuppressive chemotherapy during cancer treatment (Low and Rotstein, 2011). The most clinically significant invasive opportunistic fungal pathogens belong to one of the four groups: *Aspergillus*, *Candida*, *Cryptococcus* and *Pneumocystis*, with the first two being the most important of all fungal pathogens (Köhler et al., 2017). Currently used antifungals include three major classes of drugs with different mechanisms of action: polyenes (disrupt fungal membranes), azoles (inhibit ergosterol biosynthesis), and echinocandins (inhibit synthesis of cell wall β-glucan) (Odds et al., 2003). However, all current antifungals suffer from inefficiency, toxic side effects, drug-drug interactions and developing drug-resistance (Arendrup, 2014; Cuenca-Estrella, 2014; Fairlamb et al., 2016). In addition, since 2001, no new classes of antifungals have been approved. This emphasizes the urgent and critical need for developing a novel generation of antifungals.

Pharmacological therapies for various bacterial or viral infections based on natural, mostly herbal, agents are among the most current therapeutic trends in medicine (Pan et al., 2013; Li and Weng, 2017). The antifungal activity of a large number of natural products is well documented (Di Santo, 2010). The most well-known structures exhibiting fungicide activity are polyenes, oligopeptides, terpenoids and macrolides, while different other natural products like flavonoids, alkaloids and phenolic acids have been also reported (Tasleem et al., 2009; Teodoro et al., 2015). Olive (*Olea europaea*), extracts has been reported for their antimicrobial and bacteriostatic activity since 1970 (Walter et al., 1973). Mild antifungal activity has been also described for olive leaf extracts (Korukluoglu et al., 2008) and the major olive phenolic compound oleuropein (Zorić et al., 2016). Recently, plant extracts and chemically synthesized related analogs from olive have also shown an antiprotozoan activity (Belmonte-Reche et al., 2016; Koolen et al., 2017). Hydroxytyrosol (HT), one of the major phenolic compounds in olive oil, has recently received particular attention because of its radical-scavenging, antiproliferative, proapoptotic and anti-inflammatory activities, which seem to have a counteractive effect on carcinogenesis and other cellular malfunctions in animal trials and *in vitro*. Additionally, recent evidence has shown that HT and its analogs might be promising antibacterial, antiviral or antiprotozoan agents (Manna et al., 2005; Fernandez-Bolanos et al., 2008, 2012; Chillemi et al., 2010; Koolen et al., 2017; Thielmann et al., 2017; Robles-Almazan et al., 2018). However, no systematic effort has been invested in search of novel antifungals based on HT, except from some reports concerning yeast species (Pereira et al., 2007; Zoric et al., 2013), or HT analogs.

Based on some preliminary tests of our lab that showed a moderate antifungal effect of HT and some HT analogs on *Aspergillus nidulans*, here we systematically synthesize and test the antifungal activities of an extended series of novel HT analogs. The rationale of their synthesis was based mostly on the possible effect of the length and saturation of the fatty acid chain, and the substitution of the α-carbon of the HT side chain. We show that several of the synthesized HT analogs have a very potent and broad antifungal activity against major fungal pathogens, such as *A.* *fumigatus*, *A.* *flavus*, *Fusarium* *oxysporum*, *A.* *nidulans* and *Candida* *albicans*. Importantly, we reveal that the antifungal activity of HT analogs is directly related to its rapid destructive effect on fungal plasma membranes, which in turn justifies why resistance mutants could not be isolated. Our work is expected to open the way for developing a new class of highly potent novel antifungals.

## Results

### Chemical Synthesis of HT Analogs

**FIGURE 1:**
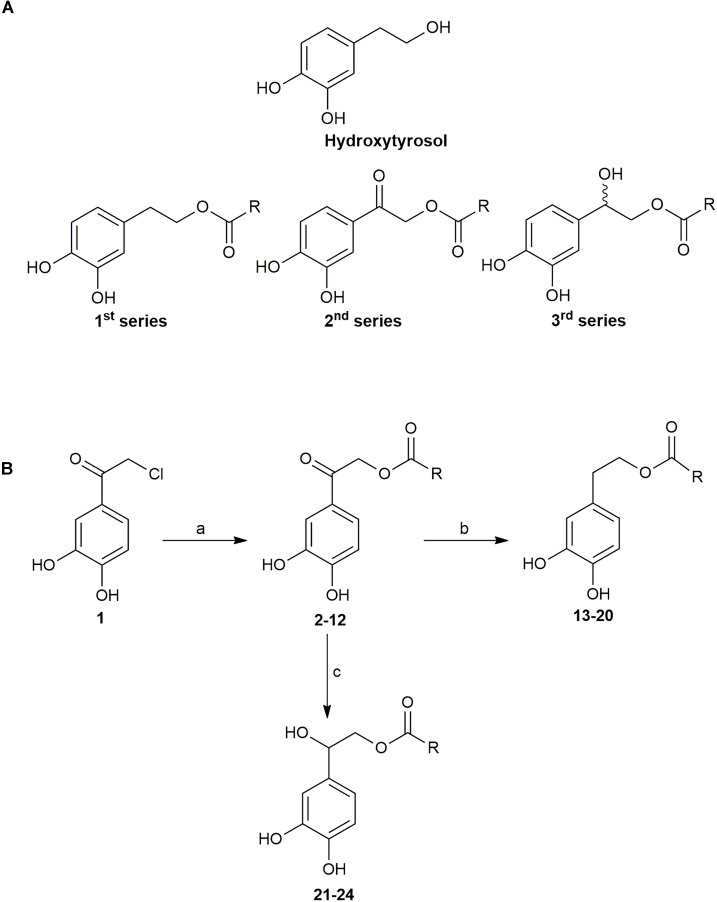
**(A)** Structures of Hydroxytyrosol (HT) and general formula of the compounds **(B)** Reactions and Conditions: a) sodium salt of suitable acid, DMF, 70°C; b) Et_3_SiH, CF_3_COOH, r.t.; c) H_2_, 10% Pd/C, t-BuOH, 50 psi, r.t.

Twenty three analogs of HT were synthesized as described in detail in the section “Materials and Methods” and in Supplementary Material (Supplementary Figure S1). The new compounds are lipophilic esters of HT bearing modifications on the α-carbon of the catechol side chain. More precisely, the new compounds are categorized in 3 different series (Figure 1A). The compounds of the second series are simple esters of HT in the aliphatic hydroxyl group, whereas the compounds of the first series possess a carbonyl group on the α-carbon of the catechol side chain. The compounds of the third series possess a hydroxyl group on the α-carbon of the catechol side chain. In brief, chloride **1** was treated with the sodium salt of the appropriate acid to afford the required keto esters **2-12** (Figure 1B). The majority of the acids are commercially available, though the acids for the preparation of ester **9** was synthesized through a Wittig reaction of methyl 4-methylene cyclohexane carboxylate. Treatment of the previous compounds with triethylsilane in trifluoroacetic acid, provided the desired, fully reduced, lipophilic esters **13-20**. It is noteworthy that this method was successful in yields up to 80%, with high reproducibility and scale up to grams. Finally, partial reduction with hydrogenation over Pd/C, provided the hydroxyl derivatives **21-24**, as racemic mixtures.

### HT Analogs Show High Potential as Antifungals

The 23 chemically synthesized analogs of HT were tested for their antifungal potential against *A. nidulans*, *A. fumigatus, A. flavus, C. albicans* and *Fusarium oxysporum*, which are important fungal pathogens of animals and/or plants. The rationale for choosing *A. nidulans* was also based on its unique amenability as a model system for genetic and functional analysis, rather than its pathogenic profile, which would allow the investigation of the molecular mechanisms underlying of the antifungal action of HT analogs. All synthesized HT analogs were tested as described in Materials and methods. We tested all analogs on solid minimal media at physiological and optimal pH ranges (5.5–6.8) and temperatures (25–37°C). The effect on *C. albicans* was also recorded in liquid fresh cultures at their logarithmic phase of growth. Importantly, similar results were obtained at the different pH tested and on complete media or minimal media. Notably, recorded apparent antifungal activities were significantly higher at 37°C compared to 25°C (see also later).

**FIGURE 2:**
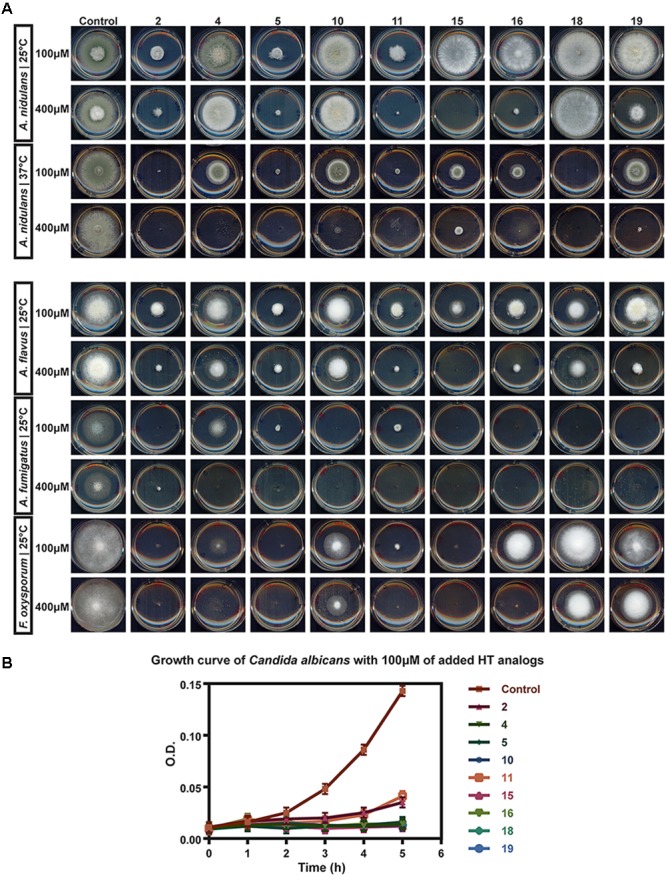
*In vivo* evaluation of HT analogs as antifungals **(A)** Growth tests showing the antifungal activity of certain HT analogs against pathogens *A. flavus A. fumigatus, F. oxysporum* and *A. nidulans.* Growth on two concentrations of HT analogs for each microorganism is shown. **(B)** Growth curve of *C. albicans.* O.D stands of Optical Density at 600 nm of liquid cultures recorded hourly. Analogs were added to the cultures at 200 μM. Control stands for cultures were only DMSO solvent was added in the cultures, at the same concentrations as the analogs.

**FIGURE 3:**
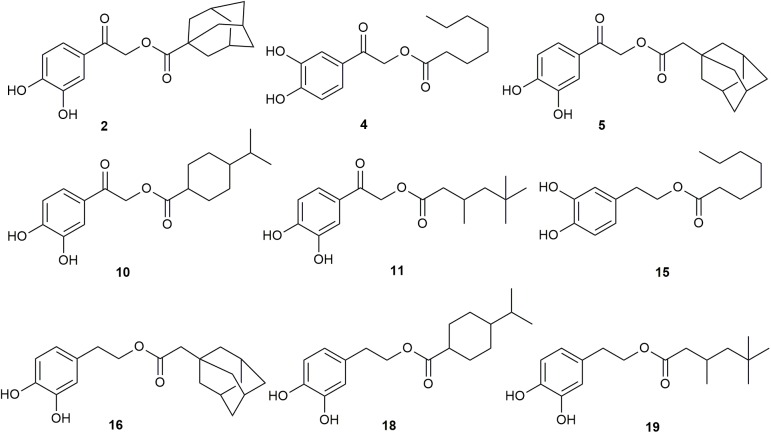
Specific HT analogs showing strongest antifungal activity.

Figure 2 and Table 1 highlight our findings and reflects the outcome obtained through at least three independent growth tests that showed practically identical results. Nine HT analogs (**2**, **4**, **5**, **10**, **11**, **15**, **16**, **18** and **19**), shown in Figure 3 had strong antifungal activity against *A. nidulans* at 37°C, mostly evident at 400 μM, and six of them (**2**, **5**, **11**, **15**, **16** and **19**) were also very active at 25°C. Most of the same nine analogs also had strong antifungal activity against other fungi tested, at 25°C and 37°C (Figure 2A, Table 1 and test not shown). In particular, all nine analogs were extremely toxic to *A. fumigatus*, leading to total or extremely severe inhibition of growth at 100 μM. *A. flavus* proved to be the most resistant fungus among those tested, but still several analogs were highly inhibitory for its growth (**2**, **5**, **11**, **15**, **16** and **19**). Best antifungal agents against *F. oxysporum* proved to be analogs **2**, **5**, **11** and **15**. All analogs, at 100 μM, severely inhibited *C. albicans* growth in liquid cultures (Figure 2B) or on solid minimal media (not shown) at 37°C. When liquid cultures were left to grow for more than 24 h after the initial addition of HT analogs, growth *C. albicans* resumed in several cases, but not in the presence of analogs **4** or **15** (Supplementary Figure S2). This indicates that these analogs had the strongest cytotoxic effect or that these compounds were the most stable under the relative experimental conditions. Overall, several synthesized analogs of HT have a dramatically increased antifungal activity, compared to the “mother” natural compound (HT), and importantly, the most of active of them are comparable to Amphotericin B (Supplementary Figure S3). The rationale of this latter comparison becomes apparent later.

**Table 1:** Approximate concentrations of HT analogs that lead to 50% reduction of fungal growth (*IC*_50_).

	HT	2	4	5	10	11	15	16	18	19
*A. nidulans* 37^o^C	>400	∼50	<100	∼50	∼100	∼50	< 100	<100	∼50	∼100
*A. nidulans* 25^o^C	>400	<100	<200	<100	<200	∼100	< 200	<200	< 200	< 200
*A. fumigatus*	>400	∼50	<100	∼50	∼50	<100	<50	<100	∼50	∼50
*A. flavus*	>400	<100	>100	<100	>100	<100	<100	∼100	∼100	>100
*F. oxysporum*	>400	<50	<50	<50	∼100	<50	<50	<200	<200	<200
*C. albicans*	>400	∼100	∼100	∼100	∼100	∼100	∼100	∼100	∼100	∼100


*IC_50_ values shown correspond to the approximate μM concentration of the compounds that reduce the diameter of colony growth by 50% after 4–6 days at 37°C, on standard agar minimal media. IC_*50*_ are estimated by testing growth in the presence of a range of μM concentration (i.e., 0, 50, 100, 200, 400, 500, and 1000 μM). The values shown were estimated by at least three independent experiments, which showed no significant variation (*SD* = 20 ± 5%). For one of the most active analogs, **15**, additional experiments performed at a lower concentration range (5, 10, 20, 50, and 100 μM), led to a more precise estimation of IC_*50*_, which corresponds to 17 μM. Most analogs led to total inhibition of growth at 100–400 μM against all fungi tested (see Figure 2).*

### Antifungal HT Analogs Show Variable Antibacterial Activities

**FIGURE 4:**
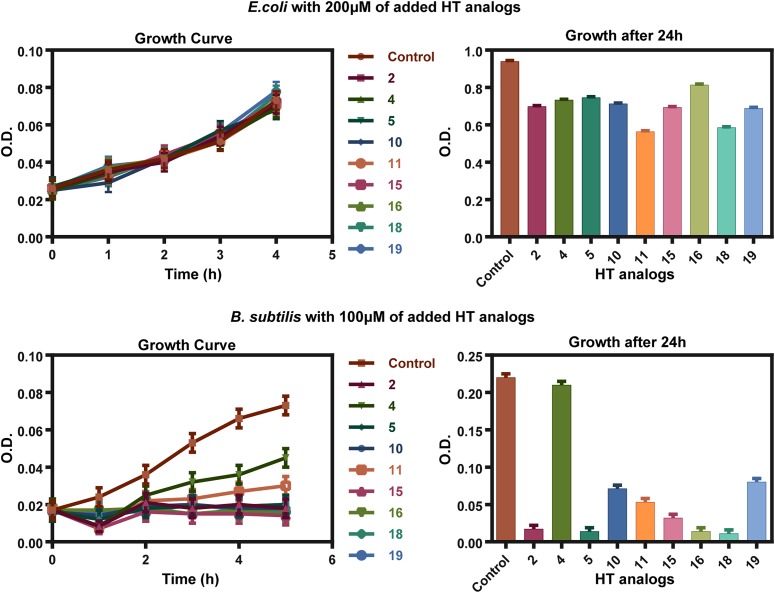
Antibacterial action of HT analogs against *E. coli* and *B. subtilis.* Growth curves on the left show O.D. values recorded hourly at 600 nm. Column bar graphs on the right show growth after 24 h after the HT analogs addition, at 600 nm. Control stands for samples where only the solvent DMSO was added at a concentration identical to the one used for the dissolved analogs.

The HT analogs with the highest antifungal activity were also tested, at a range of 100–200 μM, in fresh exponentially growing bacterial cultures in order to gain further insight on their mode of action and as a first step for investigating their potential use as broad range antimicrobials. Figure 4 shows the results obtained with *E. coli* and *B. subtilis*, as typical representatives of G- and G+ bacteria. All analogs were highly toxic to *B. subtilis* at 100 μM, but not at all to *E. coli.* *Pseudomonas* species showed differential growth behavior, with *P. aeruginosa* being fully resistant, but *P. fluorescens* highly sensitive at 200 μM (Supplementary Figure S4). Additional bacterial species were also tested (*Klebsiella, Enterococcus Staphylococcus, Streptococcus* and other *G- enterobacteria*) showing varying degrees of sensitivity to HT analogs, but this will be reported elsewhere as the present work is directed toward the discovery of novel antifungals.

### Antifungal HT Analogs Disrupt the Structure and Function of *A. nidulans* Plasma Membrane

**FIGURE 5:**
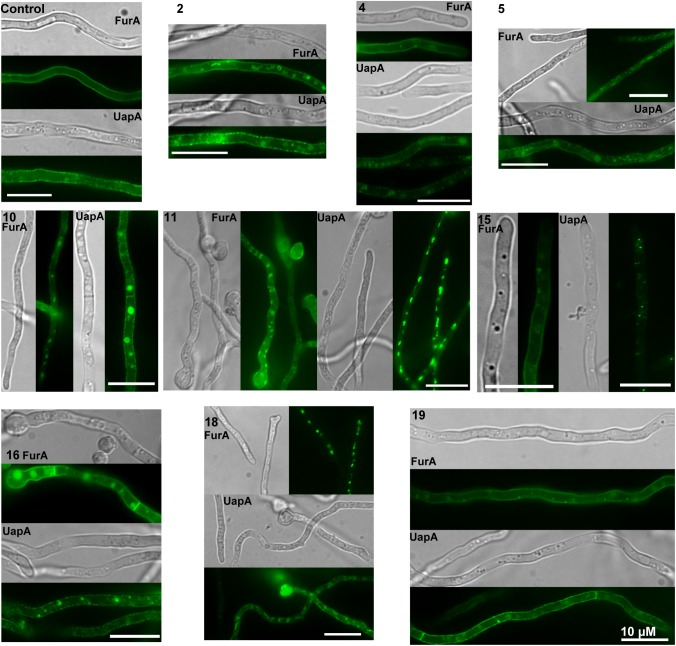
Effect of HT analogs on *A. nidulans* plasma membrane and transporter-mediated uptake of xanthine. Epifluorescence *in vivo* microscopy showing the effect of HT analogs (37.5 μM) on the plasma membrane of *A. nidulans*. The picture shows young hyphae of strains expressing functional GFP-tagged FurA or UapA transporters as PM molecular markers. FurA expression is stronger than that of UapA, due to transcription via a strong constitutive promoter (*gpdA_p_*), while UapA is transcribed by its native, relatively weak, promoter. Notice that upon addition of most analogs cortical fluorescent labeling is reduced with concurrent appearance of cytosolic fluorescent foci, which represent membrane aggregates. Scaleσ shown are 10 μM.

The non-dependence of the antifungal action of the HT analogs on whether minimal or complete media are used, or the pH range, when this was kept within limits proper for fungal growth, suggested that these compounds might be either taken up by non-facilitated diffusion, or exert their activity directly without the need to enter the cell (i.e., on the cell wall or the plasma membrane). The relatively increased activity observed at 37°C vs. 25°C in the case of *A. nidulans* (see Figure 2) does not distinguish between the two possibilities, as increased membrane fluidity at a higher temperature would favor diffusion, as well as, binding of hydrophobic HT analogs in specific lipids of the fungal membrane. Thus, to investigate this issue directly we followed the effect of all antifungal HT analogs on the microscopic morphology and the plasma membrane (PM) of *A. nidulans* hyphae, using Brightfield and Epifluorescence microscopy, respectively. For investigating the effect on the plasma membrane we used strains expressing two GFP-tagged plasma membrane transporters, namely the UapA uric acid-xanthine transporter (Pantazopoulou et al., 2007) or the FurA allantoin transporter (Krypotou et al., 2015). Figure 5 summarizes our results. Most HT analogs, when added at final concentration as low as 37.5 μM, for 0–30 min, had a rapid and prominent effect on plasma membrane integrity, reflected in dramatic reduction of transporter-associated peripheral GFP fluorescence signal and concomitant appearance of static, non-cortical cytosolic membrane fluorescent aggregates (most evident with analogs **2**, **5**, **10**, **11**, **16** or **18**). The great majority of these cytosolic fluorescent aggregates did correspond to vacuoles or other known endomembrane compartments, as evidenced by CMAC or FM4-64 staining (not shown). Under brightfield light we did not notice any dramatic modification that would be compatible with disruption of the cell wall or overall hyphae morphology (see also Supplementary Figure S5), despite a visible increase in vacuole number and size. These effects were practically immediate, becoming evident in 1–5 min, which also somehow excludes the idea that HT analogs act primarily by metabolic inhibition of an enzyme. In general, the relative strength of the detrimental effect of the different HT analogs on the PM was variable, with some analogs leading to total apparent disintegration, while others led to significant but not total, of the PM in 10 min. The concentration used to test the analogs was 37.5 μM in order to avoid any effect of DMSO (the solvent used) on the stable localization of transporters in the PM, as we have noticed that DMSO concentrations>50 μM elicit a degree of endocytic turnover for most transporters studied in our lab (unpublished observations).

**FIGURE 6:**
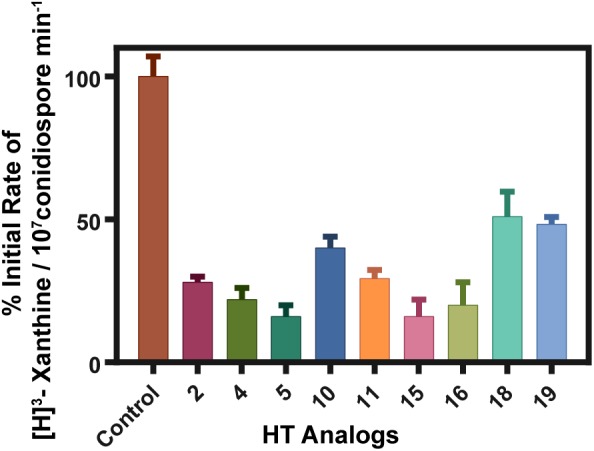
Effect of HT analogs on transporter-mediated uptake of xanthine. % of initial uptake rates are shown as recorded in a standard *A. nidulans* strain. Analogs were added at final concentration of 100 μM 10 min prior to uptake measurements. Control stands for a sample where only the solvent DMSO was added at a concentration identical to the one used as a solvent in the analog samples. Control values were arbitrary taken as 100% rate. The results shown represent averages if three independent assays with SD<15%.

To further examine the nature of the effect of HT analogs on the PM, we also performed direct transport assays of radiolabeled metabolites, imported by specific transporters, in the presence of excess analogs. Transport assays used measure initial uptake rates (at 60 s) of radiolabeled substrates in germinated conidiospores (Krypotou and Diallinas, 2014). In particular, we tested the uptake of radiolabeled xanthine, which is specifically transported by two uptake systems, the UapA (∼70%) and UapC (∼30%) transporters (Pantazopoulou and Diallinas, 2007; Krypotou and Diallinas, 2014). Results are summarized in Figure 6. HT analogs **2**, **4**, **5**, **10**, **11, 15** and **16** reduced xanthine uptake to ∼20–40% of the control sample (only DMSO added), whereas the rest (**18** and **19**) showed less inhibitory effect. Overall, these results strongly suggested that most HT analogs tested had a rapid negative effect on fungal transport systems. Given that the relevant xanthine transporters, UapA and UapC, as most fungal transporters, are H^+^ symporters, our results can in principle be explained by two scenarios; either the analogs lead to rapid disorganization of the PM, or they led to a rapid depolarization of the membrane, acting as direct H^+^ gradient uncouplers. However, the latter case seems unlikely, or secondary, under the light of the microscopic analysis shown in Figure 5, which directly confirmed the dramatic effect of all HT analogs tested on the PM within some minutes after their addition to the fungal cultures. Additionally, the strength of inhibition of transporter-mediated xanthine uptake by the different HT analogs tested was in good agreement with the results obtained following their effect on PM integrity (Figure 5) and their *in vivo* antifungal activity (Figure 2). We thus conclude that the direct target of HT analogs is disruption of the fungal PM integrity and function.

### Lack of Resistance to HT Analog Antifungal Action

Based on our findings that showed that HT analogs act directly and rapidly on the PM, we presumed that resistance to this novel type of antifungals will be infrequent if any, similar to the case of other antimicrobials that target directly the periphery of microbes, such as amphotericin B (plasma membrane) or Echinocandins (cell wall) (Cuenca-Estrella, 2014). To test this, we performed several experiments of standard u.v. or transposon-driven mutagenesis as described in Materials and methods of an appropriate *A. nidulans* strain (i.e., one carrying the Minos transposable element (Evangelinos et al., 2015)), and tried to select mutant colonies resistant to 400 μM of analog **15**. We did not manage to isolate any resistance colony, even in cases were lethality of u.v. mutagenesis was 93.7%. This apparently negative result is fully compatible with HT analogs targeting the PM of *A. nidulans*.

## Discussion

Here, we showed that specific chemically synthesized HT analogs possess broad and strong antifungal activity due to an immediate primary effect on the fungal plasma membrane. We do not, however, exclude that the HT analogs also have secondary cytotoxic targets (e.g., cell wall or enzymes), mostly in other microorganisms, which we did not test directly here. An additional finding classifies this specific set of compounds as very promising novel antifungals; lack of detectable fungal (*A. nidulans*) resistance due to mutations. The lack of obtaining spontaneous or mutation-induced resistance to an antifungal is a strong indication that the primary site of action of this antifungal is a molecule in the periphery of fungal cells, most probably a specific lipid of the PM. This is in full agreement with the effect detected herein of the HT analogs on the PM. Moreover, the lack of resistance resembles cases of other antifungals targeting the plasma membrane, such as amphotericin B, where only infrequent resistance is obtained due to mutations affecting the level of ergosterol and phospholipids in the membrane (Cuenca-Estrella, 2014; Fairlamb et al., 2016). Finally, an other aspect concerning the promising future of present HT analogs as novel antifungals is the observation that in preliminary tests analogs **15** and **16**, which showed high antifungal activity, showed no toxicity against a mammalian cell line (see Materials and methods). Overall, our results open the way for the rigorous development of a novel, efficient and maybe safer class of antifungals. Additionally, the antifungal HT analogs were also shown to also possess antimicrobial activity against specific G+ bacteria. This is an interesting, albeit rather preliminary, observation that we want to exploit in the future as it might give us information on the exact mechanism of action of interaction of these compounds with specific membrane lipids.

In several previous studies, HT has been shown to have variable antibacterial activity at relatively high concentrations, in the range of 1–4 mM (Capasso et al., 1995; Bisignano et al., 1999; Medina et al., 2006, 2007; Tafesh et al., 2011; Medina-Martínez et al., 2016). However, bacterial growth was never fully inhibited and a general conclusion on the effects of HT on Gram-positive or Gram-negative species could not be made (Medina-Martínez et al., 2016). To our knowledge, the only investigations regarding the antifungal potential of HT are those against medically important *Candida* spp., which showed minimal growth inhibitory values ranging between 633 μM and 40 mM (Pereira et al., 2007; Zoric et al., 2013). Notably, fluorescent dye-exclusion based studies with *C. albicans* revealed a membrane associated antifungal mechanism at sub-inhibitory concentrations (Zoric et al., 2013), in line with results presented herein. In the past, HT-derived compounds have been synthesized aiming at analogs with a better hydrophilic/lipophilic balance (HLB) to increase their cellular uptake and thus enhance their antioxidant or other unknown activities (Grasso et al., 2007; Bernini et al., 2012, 2015). However, no previous studies evaluating synthesized HT analogs as antifungal agents have been reported. HT derivatives have only recently been shown to have a very promising antitrypanosomal and antileishmanial activity (Belmonte-Reche et al., 2016). IC_50_ values against *Trypanosoma brucei* for HT decanoate ester and HT dodecanoate ester were 0.6 and 0.36 μM, respectively. This represented a significant 79–132 fold improvement in activity compared to HT. Focusing on structure-activity relationships, the authors found via rational design several more HT analogs to have targeted cytotoxic activity against *T. brucei* with IC_50_ values in the low micromolar range. They concluded that the di-ortho phenolic ring and medium size alkyl chain are essential for activity, whereas the nature of the chemical bond among them seems less important. Importantly, antiprotozoan HT analogs displayed a high selectivity index against MRC-5, a non-tumoral human cell line, which is in line with the targeted antimicrobial specificity we also found for the HT antifungal analogs reported here. Thus, HT is indeed a highly promising mother compound to develop novel broad range antimicrobial agents.

Several of the new compounds described here showed varying degrees of antifungal activity against the tested pathogens, nevertheless compounds **2**, **5**, **11**, **15**, **16** and **19** possessed the strongest activity, clearly higher than the mother compound HT. All the active compounds belong to the first and second series, while compounds of the third series (**21**, **22**, **23**, **24**; see Supplementary Figure S1) were non-active. The carbonyl substituted compounds of the first series, and especially the alkyl-substituted analogs **2**, **5** and **11,** were among the most potent compounds, suggesting that the substitution on the α-carbon is crucial for the activity. It seems that the presence of the hydroxyl group, is incompatible with activity, whereas the carbonyl group increases the antifungal capacity, probably by increasing the acidity of the catechol moiety. Notably also, all compounds in this study with an aromatic substitution (**3**, **6**, **14** and **22**; see Supplementary Figure S1) showed no activity against the tested pathogens, while the alkyl substituted compounds of first and second series were shown to be potent antifungals. The role of the double bond of the alkyl side chain is detrimental for the activity (see Supplementary Figure S1). Additionally, the role of the branching is not clear since the branched alkyl chained compounds **2**, **5**, **10**, **11**, **16**, **18** and **19** showed potent antifungal activity, whereas compounds **9**, **12**, **13** and **20** seem to be inactive. Nevertheless, our results demonstrate that only the alkyl substituted compounds of first and second series are active against the tested pathogens, with the most effective being those with an alkyl chain of 6 to 10 carbons. These results are also in agreement with the ones against *Trypanosoma brucei* for HT decanoate ester and HT dodecanoate ester (Belmonte-Reche et al., 2016).

According to recent data, alkyl gallates and other amphiphilic phenols, compounds possessing similar structure to the HT **analogs** presented here, have significant antifungal activity (Kozubek et al., 2001; Kubo et al., 2001b). It is suggested that their potency may result from the interaction with cell wall components (phospholipids, etc.); therefore, their activity is due partly to the ability of these molecules to function as nonionic surfactants (surface-active compounds), disrupting the fungi membrane. The mechanism by which surfactants exert their activity is not entirely understood, it is, however, hypothesized that, due to their amphiphilic character, they are inserted into the fungi lipid bilayer (possibly in other microbial membranes also), where they elicit an immediate destruction effect (Kubo et al., 2001a; Koziróg et al., 2018). In that aspect, the amphiphilic character of the HT analogs presented in this work and the experimental evidence for rapid destruction of the fungus membrane allow us to reasonably assume that they act primarily as nonionic surfactants, though other additional molecular interaction could account for the final effect. However, specific structural characteristics are needed for optimal antifungal activity strongly depended from a balance between the polar hydrophilic and the non-polar hydrophobic part of the molecules. The aliphatic ester side chain (hydrophobic tail) is assumed to interfere with the cell membrane, while the acidity of the catechol group (hydrophilic head) seems to be essential for the antifungal activity. The initial contact with the lipid bilayer should involve the electrostatic interaction of the catechol system with the negatively charged phosphate groups of the membrane; thus the increased acidity of the hydroxyl groups due to the carbonyl substitution might be essential for activity. The divergence of the activity in relation to the structure of the alkyl tail indicates its vital role in the action of the compounds. The length (6 to 10 carbons) and increased volume because of alkyl substitutions are fundamental for optimum activity, whereas the substitution of the α-carbon is critical for the electrostatic interaction of the hydrophilic head. The rationale for the role of the side alkyl chain is not entirely understood, and further research is needed in order to design more effective antifungal agents. Nevertheless, the fact that the HT analogs, very likely, targets the extra- cytoplasmic region could explain, as mentioned before, the lack of resistance of the described molecules.

Although recent technological advances made accessible unprecedented tools for drug discovery the development of new molecular scaffolds with demonstrated pharmacological properties is becoming slower and more expensive. Our study shows once more that the natural product chemical space provides the basis of successful design of new small-molecular-weight molecules with excellent pharmacological properties. As HT has been shown to undergo rapid oxidation the antifungal HT analogs described here will, however, need to be tested *in vivo* in mouse models and evaluated in respect to their stability or synergistic or antagonistic effects with other antifungals.

## Materials and Methods

### Synthesis of HT Analogs

Chemical synthesis of HT analogs is described in detail in Supplementary Material. HT analogs were prepared in DMSO and aliquots and kept at -20°C. For experiments, the final concentration of DMSO in the medium was<0.1% (v/v) and the controls received DMSO only.

### Fungal and Bacterial Strains and Growth Media

Standard “wild-type” fungal strains of *A.* *nidulans* (FGSC A4)*, A. fumigatus* **(**Af293)*, A. flavus* (NRRL3357), *F. oxysporum* (F3; (Christakopoulos et al., 1996)) and *C. albicans* clinical isolate from the Mycetotheca ATHUM collection of Athens University^1^ were used. The *A. nidulans* used contains a mutant allele (*veA1*) at the *velvet* locus, and a standard vitamin auxotrophy (*pabA1* for para-aminobenzoic acid requirement). All other Aspergilli used correspond to standard strains used for genome sequencing^2^. For *in vivo* epifluorescence microscopy, strains expressing GFP-tagged functional UapA (Pantazopoulou et al., 2007) or FurA (Fernandez-Bolanos et al., 2012) transporters were used. Standard Aspergillus Minimal and Complete Media (MM and CM) were used for growth of all fungi^3^. The nitrogen source used in MM was 10 mM NaNO_3_. Standard bacterial strains, coming from an in-house stock, *of E. coli (DH5a), P. aeruginosa, P. fluorescens, Klebsiella sp., B. subtilis* and *S. aureus* were used. Luria-Bertani medium was used for growth of bacterial strains.

### *In vivo* Evaluation of HT Analogs as Antifungals

Fungal strains of *A.* *nidulans, A. fumigatus, A. flavus, F. oxysporum* and *C. albicans* were tested for their sensitivity/ resistance to different concentrations. Fungal spores of each strain were used to centrally inoculate a series of 35mm petri dishes containing Minimal Media (MM) with NaNO_3_ as nitrogen source, in the presence or absence of various HT analog concentrations dissolved in DMSO. Controls without HT analogs contained solely DMSO. The final concentration range tested for toxicity of analogs in this work was 0–1000 μM. Figures shown highlight results using concentrations of 100 or 400 μM. Growth was followed after for 4–6 days, at 25 or 37°C and different pH. Approximate concentrations leading to 50% reduction in the radial diameter of the growing colonies, (*IC*_50_), after 2–4 days (depending on the fungus) were recorded for each analog. Approximate concentrations leading to no evident fungal growth were also recorded. For the non-filamentous fungus *C. albicans* we performed sensitivity tests in both liquid and solid cultures. For solid cultures testing, cells from a fresh liquid culture (O.D._600nm_ = 0.5 at 10 nm) were streaked on standard CM containing 100–400 μM of HT analogs, and incubated for 2 days at 37°C. For liquid culture testing, HT analogs were added, at 100–400 μM, at the start of the exponential phase (O.D._600nm_ = 0.5 at 10 nm) of a *C. albicans* culture and O.D._600nm_ measurements were recorded hourly for the next 6 h, and after 24 h. All final values shown in this article are averages of at least three independent experiments with no significant SD variation (e.g., <20%).

### *In vivo* Evaluation of HT Analogs as Antibacterial Agents

Standard bacterial strains *of E. coli (DH5a), P. aeruginosa, P. fluorescens, Klebsiella sp., B. subtilis* and *S. aureus* were tested for their sensitivity/resistance to different concentrations of compounds, which proved to act as antifungals. These tests were carried out either in solid or liquid LB media, in the presence or absence of 100–500 μM of HT analogs. Tests on solid media were performed by recording single colony growth after bacterial streaking. Tests in liquid media were performed by recording O.D._600nm_ values (10 nm) after addition of 100–200 μM of compounds in fresh exponentially growing bacterial cultures (O.D._600nm_ = 0.2–0.4), and comparing these values to control cultures with no analogs. All final values shown in this article are averages of at least three independent experiments (SD variation<15%).

### *In vivo* Epifluorescence Microscopy

Samples for standard inverted epifluorescence microscopy of *A. nidulans* strains were prepared as previously described (Martzoukou et al., 2017). In brief, germlings were incubated in sterile 35 mm μ-dishes, high glass bottom (*ibidi*, Germany) in 2 ml liquid MM with NaNO_3_ as nitrogen source and the necessary vitamin supplements for 20 h at 25°C. DMSO (0.1%) or HT analogs (final concentrations tested 37.5 or 100 μM) dissolved in DMSO were added in samples under the microscope. Control samples where only DMSO was added (0.03–0.01%) were also evaluated. Images were taken before and immediately after the addition of the analogs (or DMSO) and for a period of up to 30 min. The strains used express the UapA or the FurA transporter as protein fluorescent markers specific for the plasma membrane (Pantazopoulou et al., 2007; Krypotou et al., 2015). Calcuofluor white staining of cell wall was as described in Martzoukou et al. (2017). Images were obtained with an AxioCa m HR R3 camera using the Zen lite 2012 software. Contrast adjustment, area selection and color combining were made using the Zen 2012 software. Images exported as tiffs were annotated and further processed in Adobe Photoshop CS4 Extended version 11.0.2 software for brightness adjustment, rotation and alignment.

### Transport Measurements

Kinetic analysis-[^3^H]-xanthine (21.6 Ci/mmol, Moravek Biochemicals, CA, United States) uptake in MM was assayed in germinating conidiospores of *A. nidulans* concentrated at 10^7^ conidiospores/100 μL, at 37°C, pH 6.8, as previously described (Krypotou and Diallinas, 2014). Initial velocities were measured at 1 min of incubation with concentrations of 0.2–2.0 μM of [^3^H]-xanthine at the polarity maintenance stage (3–4 h, 130 rpm).

### Mutagenesis

Two U.V. mutagenesis experiments, with lethality rates of 84.1 and 93.7%, were performed at a standard distance of 20 cm from an Osram HNS30 UV-B/C lamp. 10^9-10^ conidiospores of a standard wild-type *A. nidulans* strain or a strain possessing a dual transposition system based on the *Minos* element (Evangelinos et al., 2015) were irradiated for 4 min and subsequently plated on MM plus nitrate medium containing 400 μM of the HT analog 15. No colonies appeared after 1-week incubation at 25°C.

### Toxicity in a Mammalian Cell Line

N2A mouse neuroblastoma cells were used, in a standard MTT assay (Berridge et al., 2005), to test the whether antifungal HT analogs elicit cytotoxicity in a standard mammalian cell line. Analogs 15 or 16 were tested at two concentrations (100 or 400 μM) as described in materials and methods. The N2A mouse neuroblastoma cells were grown in Dulbecco’s Modified Eagle’s Medium (DMEM) that contained 10% fetal bovine serum, 1% of penicillin and streptomycin in 96-well plates at a density of 15,000 cells/well. A standard MTT assay was used to assess cell metabolic activity in the absence (addition of solely DMSO) and presence of HT analogs (Berridge et al., 2005). The cultures were grown for 6 days at 37°C with 5% CO_2_. Then the medium was changed to one containing 100 or 400 μM of 15 or 16 and incubated for 24 h at 37°C with 5% CO_2_. In all cases the final concentration of DMSO was≤0.1%. 20 μl of the dye MTT (2.5 mg/ml MTT in PBS) was added to each well and incubated for 4 h. The resulting formazan dye was extracted with 100 μl isopropanol/HCl (100 ml isopropanol + 833 μl HCl) and the absorbance was measured spectrophotometrically at a wavelength of 545 nm. Statistical analysis: All experiments were repeated three times. One-way ANOVA with Bonferroni’s Multiple Comparison Test was used to evaluate the statistical significance of the differences. Statistical significance was defined as *p*<0.05.

## Author Contributions

GD performed and designed the biological experiments, analyzed the results and wrote the manuscript. NR performed the biological experiments and made relevant figures. VT and IZ performed the biological experiments. IK and AK performed the chemical experiments. EM and AS analyzed the results. IKK designed the chemical synthesis, analyzed the results and wrote the manuscript.

## Conflict of Interest Statement

The authors declare that the research was conducted in the absence of any commercial or financial relationships that could be construed as a potential conflict of interest.

## Footnotes

1 http://mycetotheca.biol.uoa.gr/

2 http://genome.jgi.doe.gov/programs/fungi/index.jsf

3 http://www.fgsc.net
