# Supplementary material for: Hydroxytyrosol (HT) Analogs Act as Potent Antifungals by Direct Disruption of the Fungal Cell Membrane
Source: Front Microbiol. 2018 Nov 6;9:2624. doi: 10.3389/fmicb.2018.02624 (PMC6232300; doi:10.3389/fmicb.2018.02624)
Supplement: Supplementary file 1 [file Data_Sheet_1.docx]

Supplementary Material

Hydroxytyrosol (HT) analogs act as potent antifungals by direct disruption of the fungal cell membrane

**George Diallinas*, Nausica Rafailidou, Ioanna Kalpaktsi, Aikaterini Christina Komianou, Vivian Tsouvali, Iliana Zantza, Emmnauel Mikros, Alexios Leandros Skaltsounis, Ioannis K. Kostakis***

*** Correspondence:** George Diallinas: diallina@biol.uoa.gr

Ioannis K. Kostakis: ikkostakis@pharm.uoa.gr

**Supplementary Data**

**Experimental**

Melting points were determined on a Büchi apparatus and are uncorrected. ^1^H NMR spectra and 2D spectra were recorded on a Bruker Avance III 600 or a Bruker Avance DRX 400 instrument, whereas ^13^C NMR spectra were recorded on a Bruker Avance III 600 or a Bruker AC 200 spectrometer in deuterated solvents and were referenced to TMS (d scale). The signals of 1H and ^13^C spectra were unambiguously assigned by using 2D NMR techniques: COSY, NOESY, HMQC, and HMBC. Mass spectra were recorded with a LTQ Orbitrap Discovery instrument, possessing an Ionmax ionization source. Flash chromatography was performed on Merck silica gel 60 (0.040-0.063 mm). Analytical thin layer chromatography (TLC) was carried out on precoated (0.25 mm) Merck silica gel F-254 plates.

**General Procedure for the synthesis of compounds 2-12**

Sodium hydride (260 mg, 6.52 mmol, 60% in paraffin oil) was added at 0 °C, under argon to a solution of the appropriate acid (3.26 mmol) in dry DMF (20 mL) and the reaction mixture was stirred at room temperature for 5 min. The reaction was then cooled to 0 °C, a solution of 2-Chloro-3′,4′-dihydroxyacetophenone (0.91 g, 4.89 mmol) in DMF (2 mL) was added dropwise and the mixture was stirred at 70 °C for 4-8 h. After completion of the reaction, the volatiles were vacuum evaporated, the resulting residue was dissolved in ethyl acetate (60 mL) and washed with water (3 x 20 mL), saturated NaCl solution, dried (anhydrous Na_2_SO_4_) and evaporated to dryness. The residue was purified by column chromatography to afford the title compounds.

2-(3,4-dihydroxyphenyl)-2-oxoethyl adamantane-1-carboxylate (**2**).

The crude product was purified by column chromatography (silica gel) using a mixture of cyclohexane / ethyl acetate (4/1) as the eluent, affording the corresponding ester **2** in 73% yield.

Mp: 172-173 °C (Ethanol). ^1^H NMR (400 MHz, DMSO-*d6*) δ (ppm): 10.00 (br s, 1H, D_2_O exchang., 3’-O*H*), 9.45 (br s, 1H, D_2_O exchang., 4’-O*H*), 7.35 (d, *J* = 8.2 Hz, 1H, H-6’), 7.31 (s, 1H, H-2’), 6.84 (d, *J* = 8.2 Hz, 1H, H-5’), 5.31 (s, 2H, C*H*_2_O), 2.04-1.96 (m, 3H, C*H* _adamantyl_), 1.95-1.86 (m, 6H, C*H*_2 adamantyl_), 1.78-1.60 (m, 6H, C*H*_2 adamantyl_). ^13^C NMR (151 MHz, DMSO-*d6*) δ (ppm): 190.70 (*C*OCH_2_), 175.96 (O*C*O), 151.23 (C-4’), 145.39 (C-3’), 125.86 (C-1’), 120.98 (C-6’), 115.16 (C-5’), 114.47 (C-2’), 65.56 (*C*H_2_O), 39.99 (C _adamantyl_), 38.46 (*C*H_2 adamantyl_), 35.94 (*C*H_2 adamantyl_), 27.29 (*C*H _adamantyl_). HR-MS (ESI) m/z: Calcd for C_19_H_21_O_5_: [M1 - H]^-^ = 329.1394, found 329.1386.

2-(3,4-dihydroxyphenyl)-2-oxoethyl 3,5-dihydroxy benzoate (**3**)

The crude product was purified by column chromatography (silica gel) using a mixture of cyclohexane / ethyl acetate (1/1) as the eluent, affording the corresponding ester **3** in 64% yield.

Mp: 275-276 °C (Dec.) (EtOAc). ^1^H NMR (400 MHz, DMSO-*d6*) δ (ppm): 9.76 (br s, 4H, D_2_O exchang., 3-O*H*, 5-O*H*, 3-O*H*’, 4’-O*H*), 7.41 (d, *J* = 8.3 Hz, 1H, H-6’), 7.36 (s, 1H, H-2’), 6.89 (s, 2H, H-2, H-6), 6.87 (d, *J* = 8.3 Hz, 1H, H-5’), 6.49 (s, 1H, H-4), 5.55 (s, 2H, C*H*_2_O). ^13^C NMR (151 MHz, DMSO-*d6*) δ (ppm): 190.75 (*C*OCH_2_), 165.40 (O*C*O), 158.64 (C-3, C-5), 151.45 (C-4’), 145.55 (C-3’), 131.07 (C-1), 125.88 (C-1’), 121.19 (C-6’), 115.34 (C-5’), 114.58 (C-2’), 107.48 (C-4), 107.38 (C-2, C-6), 66.54 (*C*H_2_O). HR-MS (ESI) m/z: Calcd for C_15_H_11_O_7_: [M1 - H]^-^ = 303.0510, found 303.0500.

2-(3,4-dihydroxyphenyl)-2-oxoethyl octanoate (**4**)

The crude product was purified by column chromatography (silica gel) using a mixture cyclohexane / ethyl acetate (4/1) as the eluent, affording the corresponding ester **4** in 78% yield.

Mp: 105-106 °C (Et_2_O/n-hexane). ^1^H NMR (400 MHz, CDCl_3_) δ (ppm): 7.58 (d, *J* = 2.0 Hz, 1H, H-2’), 7.41 (dd, *J* = 8.3, 2.0 Hz, 1H, H-6’), 6.93 (d, *J* = 8.3 Hz, 1H, H-5’), 5.35 (s, 2H, C*H*_2_O), 2.53 (t, *J* = 7.6 Hz, 2H, 2-C*H*_2_), 1.76–1.66 (m, 2H, 3-C*H*_2_), 1.42–1.24 (m, 8H, 4-C*H*_2_, 5-C*H*_2_, 6-C*H*_2_, 7-C*H*_2_), 0.90 (t, *J* = 7.6 Hz, 3H, C*H*_3_). ^13^C NMR (151 MHz, CDCl_3_) δ (ppm): 191.67 (*C*OCH_2_), 174.46 (O*C*OCH_2_), 150.29 (C-4’), 143.86 (C-3’), 126.94 (C-1’), 122.43 (C-6’), 114.97 (C-5’), 114.61 (C-2’), 65.83 (*C*H_2_O), 34.02 (C-2), 31.63 (C-6), 29.06 (C-5), 28.91 (C-4), 24.85 (C-3), 22.59 (C-7), 14.05 (C-8). HR-MS (ESI) m/z: Calcd for C_16_H_21_O_5_: [M1 - H]^-^ = 293.1394, found 293.1386.

2-(3,4-dihydroxyphenyl)-2-oxoethyl 2-(adamantan-1-yl)acetate (**5**)

The crude product was purified by column chromatography (silica gel) using a mixture of cyclohexane / ethyl acetate (2/1) as the eluent, affording the corresponding ester **5** in 88% yield.

^1^H NMR (600 MHz, CDCl_3_) δ (ppm): 7.54 (d, *J* = 2.0 Hz, 1H, H-2’), 7.40 (dd, *J* = 8.3, 2.0 Hz, 1H, H-6’), 7.03 (br s, 1H, D_2_O exchang., 3’-O*H*), 6.90 (d, *J* = 8.3 Hz, 1H, H-5’), 6.40 (br s, 1H, D_2_O exchang., 4’-O*H*), 5.30 (s, 2H, C*H*_2_O), 2.26 (s, 2H, C*H_2_*CO), 1.98 (m, 3H, C*H* _adamantyl_), 1.74–1.59 (m, 12H, C*H*_2 adamantyl_). ^13^C NMR (151 MHz, CDCl_3_) δ (ppm): 191.77 (*C*OCH_2_), 172.39 (O*C*OCH_2_), 150.34 (C-4’), 143.98 (C-3’), 127.22 (C-1’), 122.56 (C-6’), , 115.10 (C-5’), 114.77 (C-2’), 65.81 (*C*H_2_O), 48.74 (*C*H_2_CO), 42.45 (*C*H_2 adamantyl_), 36.83 (*C*H_2 adamantyl_), 33.17 (C _adamantyl_), 28.77 (*C*H _adamantyl_). HR-MS (ESI) m/z: Calcd for C_20_H_22_O_5_: [M1 - H]^-^ = 343.1550, found 343.1546.

(E)-2-(3,4-dihydroxyphenyl)-2-oxoethyl 3-(4-hydroxy-3-methoxyphenyl)acrylate (**6**)

The crude product was purified by column chromatography (silica gel) using a mixture of cyclohexane / ethyl acetate (1/5) as the eluent, affording the corresponding ester **6** in 75% yield.

^1^H NMR (600 MHz, CDCl_3_-MeOD) δ (ppm): 7.62 (d, *J* = 15.9 Hz, 1H, COCH=C*H*), 7.40 (d, *J* = 2.0 Hz, 1H, H-2’), 7.38 (dd, *J* = 8.3 Hz, 2.0 Hz, 1H, H-6’), 7.03-7.06 (m, 2H, H-2, H- 6), 6.83-6.87 (m, 2H, H-5, H-5’), 6.33 (d, *J* = 15.8 Hz, 1H, COC*H*=CH), 5.30 (s, 2H, C*H*_2_O), 3.82 (s, 3H, OC*H*_3_). ^13^C NMR (151 MHz, CDCl_3-_-MeOD) δ (ppm): 193.11 (*C*OCH_2_), 168.28 (O*C*OCH), 152.48 (C-4’), 150.29 (C-4), 148.95 (C-5), 147.42 (C-3’), 146.32 (COCH=*C*H), 127.37 (C-1’), 127.29 (C-1), 124.02 (C-6’), 122.44 (C-2), 116.23 (C-5’), 115.83 (CO*C*H=CH), 115.38 (C-3), 114.44 (C-2’), 111.41 (C-6), 66.70 (*C*H_2_O), 56.32 (O*C*H_3_). HR-MS (ESI) m/z: Calcd for C_18_H_15_O_7_: [M1 - H]^-^ = 343.0823, found 343.0817.

(E)-2-(3,4-dihydroxyphenyl)-2-oxoethyl docos-13-enoate (**7**)

The crude product was purified by column chromatography (silica gel) using a mixture of cyclohexane / ethyl acetate (4/1) as the eluent, affording the corresponding ester **7** in 76% yield.

^1^H NMR (600 MHz, CDCl_3_) δ (ppm): 7.55 (d, *J* = 2.0 Hz, 1H, H-2’), 7.40 (dd, *J* = 8.3, 2.0 Hz, 1H, H-6’), 6.91 (d, *J* = 8.3 Hz, 1H, H-5’), 6.83 (br s, 1H, D_2_O exchang., 3’-O*H*), 6.26 (br s, 1H, D_2_O exchang., 4’-O*H*), 5.38–5.32 (m, 2H, C*H*=C*H*), 5.31 (s, 2H, C*H*_2_O), 2.51 (t, *J* = 7.6 Hz, 2H, COC*H*_2_), 2.04–1.98 (m, 4H, 12-C*H*_2,_ 15-C*H*_2_), 1.70 (m, 2H, 3-C*H*_2_), 1.40–1.17 (m, 28H, C*H*_2 erucic_), 0.88 (t, *J* = 7.0 Hz, 3H, C*H*_3_). ^13^C NMR (151 MHz, CDCl_3_) δ (ppm): 191.56 (*C*OCH_2_), 174.36 (O*C*OCH_2_), 150.26 (C-4’), 143.93 (C-3’), 130.06 (*C*H=*C*H), 127.25 (C-1’), 122.56 (C-6’), 115.11 (C-5’), 114.74 (C-2’), 65.92 (*C*H_2_O), 34.17 (C-2), 32.06 (C-20), 29.93 (*C*H_2erucic_), 29.86 (*C*H_2 erucic_), 29.77 (*C*H_2 erucic_), 29.76 (*C*H_2 erucic_), 29.72 (*C*H_2 erucic_), 29.68 (*C*H_2 erucic_), 29.61 (*C*H_2 erucic_), 29.47 (*C*H_2 erucic_), 29.42 (*C*H_2 erucic_), 29.28 (*C*H_2 erucic_), 27.37 (C-12, C-15), 25.02 (C-3), 22.83 (C-21), 14.26 (*C*H_3_). HR-MS (ESI) m/z: Calcd for C_30_H_47_O_5_: [M1 - H]^-^ = 487.3429, found 487.3420.

(E)-2-(3,4-dihydroxyphenyl)-2-oxoethyl penta-2,4-dienoate (**8**)

The crude product was purified by column chromatography (silica gel) using a mixture of CH_2_Cl_2_ / MeOH (100/0.75) as the eluent, affording the corresponding ester **8** in 80% yield.

^1^H NMR (600 MHz, Acetone-*d*_6_) δ δ (ppm): 7.31-7.37 (m, 2H, H-2’, H-6’), 7.18 (m, 1H, COCH=C*H*), 6.83 (d, *J* = 8.3 Hz, 1H, H-5’), 6.25–6.09 (m, 2H, COC*H*=CH, CH_3_CH=C*H*), 5.82 (m, 1H, CH_3_C*H*=CH), 1.73 (t, *J* = 5.1 Hz, 1H, C*H*_3_). ^13^C NMR (151 MHz, CDCl_3_) δ (ppm): 191.28 (*C*OCH_2_), 166.70 (O*C*OCH), 151.62 (C-4’), 146.42 (*C*H _sorbic_), 146.11 (C-3’), 140.57 (*C*H _sorbic_), 130.69 (*C*H _sorbic_), 128.05 (C-1’), 122.21 (C-6’), 119.28 (*C*H _sorbic_), 115.91 (C-5’), 115.33 (C-2’), 66.49 (*C*H_2_O), 18.69 (*C*H_3_). HR-MS (ESI) m/z: Calcd for C_14_H_13_O_5_: [M1 - H]^-^ = 261.0768, found 261.0770.

2-(3,4-dihydroxyphenyl)-2-oxoethyl 4-methylenecyclohexanecarboxylate (**9**)

The crude product was purified by column chromatography (silica gel) using a mixture of CH_2_Cl_2_ / MeOH (100/1.5) as the eluent, affording the corresponding ester **9** in 85% yield.

^1^H NMR (600 MHz, CDCl_3_) δ (ppm): 7.51 (d, *J* = 2.0 Hz, 1H, H-2’), 7.40 (dd, *J* = 8.3, 2.1 Hz, 1H, H-6’), 6.91 (d, *J* = 8.3 Hz, 1H, H-5’), 6.60 (br s, 1H, D_2_O exchang., 3’-O*H*), 6.30 (br s, 1H, D_2_O exchang., 4’-O*H*), 5.30 (s, 2H, C*H*_2_O), 4.67 (s, 2H, C*H_2_=*C, 2.67 (m, 1H, COC*H*), 2.44–2.35 (m, 2H, C*H* _cyclohexyl_), 2.14–2.07 (m, 4H, C*H* _cyclohexyl_), 1.73–1.64 (m, 2H, C*H* _cyclohexyl_). ^13^C NMR (151 MHz, CDCl_3_) δ (ppm): 191.23 (*C*OCH_2_), 175.67 (O*C*OCH), 150.08 (C-4’) ,147.60 (*C*=CH_2_), 143.91 (C-3’), 127.40 (C-1’), 122.51 (C-6’),115.09 (C-5’),114.69 (C-2’), 108.24 (C=*C*H_2_), 68.16 (*C*HCO), 65.84 (*C*H_2_O), 42.53(C _cyclohexyl_), 33.70 (C _cyclohexyl_), 30.26(C _cyclohexyl_), 25.76 (C _cyclohexyl_). HR-MS (ESI) m/z: Calcd for C_16_H_17_O_5_: [M1 - H]^-^ = 289.1081, found 289.1079.

2-(3,4-dihydroxyphenyl)-2-oxoethyl 4-isopropylcyclohexanecarboxylate (**10**)

The crude product was purified by column chromatography (silica gel) using a mixture of CH_2_Cl_2_ / MeOH (100/1) as the eluent, affording the corresponding ester **10** in 78% yield.

^1^H NMR (600 MHz, CDCl_3_) δ (ppm): δ 7.38 (d, *J* = 2.0 Hz, 1H, H-2’), 7.29 (dd, *J* = 8.3, 2.0 Hz, 1H, H-6’), 6.80 (d, *J* = 8.3 Hz, 1H, H-5’), 5.29 – 5.20 (m, 2H, C*H*_2_O), 2.46 (m, 1H, CH), 2.26 (m, 1H, CH), 2.14 – 1.98 (m, 1H, CH), 1.23 (m, 1H, CH), 1.11 (m, 1H, CH), 1.04-0.98 (m, 3H, CH_3_), 0.87 (d, *J* = 1.9 Hz, 9H, CH_3_). ^13^C NMR (151 MHz, CDCl_3_) δ (ppm): δ 192.06 (*C*OCH_2_), 173.51 (O*C*OCH_2_), 150.69 (C-4’), 144.53 (C-3’), 126.70 (C-1’), 122.13 (C-6’), 115.03 (C-5’), 114.60 (C-2’), 65.75 (*C*H_2_O), 50.61 (*C*H_2_CH), 43.58 (*C*H_2_CO), 31.06 (C), 29.96 (3xC*H*_3_), 27.03 (*C*H), 22.60 (C*H*_3_). HR-MS (ESI) m/z: Calcd for C_18_H_23_O_5_: [M1 - H]^-^ = 319.1551, found 319.1542.

2-(3,4-dihydroxyphenyl)-2-oxoethyl 3,5,5-trimethylhexanoate (**11**)

The crude product was purified by column chromatography (silica gel) using a mixture of cyclohexane / CH_2_Cl_2_ (1/10) as the eluent, affording the corresponding ester **11** in 89% yield.

^1^H NMR (600 MHz, CDCl_3_) δ (ppm): 7.38 (d, J = 2.0 Hz, 1H, H-2’), 7.29 (dd, *J* = 8.3, 2.0 Hz, 1H, H-6’), 6.80 (d, *J* = 8.3 Hz, 1H, H-5’), 5.29–5.20 (m, 2H, C*H*_2_O), 2.46 (m, 1H, C*H*), 2.26 (m, 1H, C*H*), 2.14–1.98 (m, 1H, C*H*), 1.23 (m, 1H, C*H*), 1.11 (m, 1H, C*H*), 1.04-0.98 (m, 3H, C*H*_3_), 0.87 (d, J = 1.9 Hz, 9H, C*H*_3_). ^13^C NMR (151 MHz, CDCl_3_) δ (ppm): 192.06 (*C*OCH_2_), 173.51 (O*C*OCH_2_), 150.69 (C-4’), 144.53 (C-3’), 126.70 (C-1’), 122.13 (C-6’), 115.03 (C-5’), 114.60 (C-2’), 65.75 (*C*H_2_O), 50.61 (C-4), 43.58 (C-2), 31.06 (C-5), 29.96 (*C*H_3_), 26.99 (C-3), 22.60 (*C*H_3_). HR-MS (ESI) m/z: Calcd for C_17_H_23_O_5_: [M1 - H]^-^ = 307.1550, found 307.1540.

2-(3,4-dihydroxyphenyl)-2-oxoethyl 2-cyclohexylacetate (**12**)

The crude product was purified by column chromatography (silica gel) using a mixture of cyclohexane / ethyl acetate (4/1) as the eluent, affording the corresponding ester **12** in 95% yield, as an oil.

^1^H NMR (400 MHz, CDCl_3_) δ (ppm): 6.81 (d, *J* = 8.0 Hz, 1H, H-5’), 6.75 (d, *J* = 1.8 Hz, 1H, H-2’), 6.62 (dd, *J* = 8.0, 1.8 Hz, 1H, H-6’), 4.27 (t, *J* = 7.0 Hz, 2H, CH_2_C*H*_2_O), 2.84 (t, *J* = 7.0 Hz, 2H, C*H*_2_CH_2_O), 2.19 (d, *J* = 7.1 Hz, 2H, COC*H*_2_), 1.82–1.60 (m, 6H, H-1, H-2, H-3, H-4, H-5, H-6), 1.31–1.08 (m, 3H, H-3, H-4, H-5), 0.98–0.91 (m, 2H, H-2, H-6). ^13^C NMR (151 MHz, CDCl_3_) δ (ppm): 174.33 (*C*O), 143.89 (C-3’), 142.58 (C-4’), 130.18 (C-1’), 121.07 (C-6’), 115.83 (C-5’), 115.31 (C-2’), 65.23 (CH_2_*C*H_2_O), 42.29 (CO*C*H_2_), 34.89 (*C*H_2_CH_2_O), 34.41 (C-1), 32.94 (C-2, C-6), 26.90 (C-4), 26.06 (C-3, C-5). HR-MS (ESI) m/z: Calcd for C_16_H_19_O_5_: [M1 - H]^-^ = 291.1237, found 291.1237.

**General Procedure for the synthesis of compounds 13-20**

Triethylsilane (0.312 mL, 1.96 mmol) was added dropwise to a suspension of the appropriate ester **2**-**12** (0.49 mmol) in trifluoroacetic acid (0.190 mL, 2.45 mmol), at 0 °C. The flask was sealed and the resulting mixture was stirred at room temperature for 3-6 h. After completion of the reaction, the volatiles were vacuum evaporated, the resulting residue was dissolved in ethyl acetate (40 mL) and washed with water (3 x 15 mL), saturated NaCl solution, dried (anhydrous Na_2_SO_4_) and evaporated to dryness.

3,4-dihydroxyphenethyl adamantane-1-carboxylate (**13**)

The crude product was purified by column chromatography (silica gel) using a mixture of cyclohexane / ethyl acetate (4/1) as the eluent, affording the corresponding ester **13** in 77% yield.

Mp: 151-152 °C (c-Hex). ^1^H NMR (400 MHz, DMSO-*d6*) δ (ppm): 8.78 (br s, 1H, D_2_O exchang, 4’-O*H*), 8.69 (br s, 1H, D_2_O exchang, 3’-O*H*), 6.64 (d, *J* = 7.9 Hz, 1H, H-5’), 6.61 (d, *J* = 1.5 Hz, 1H, H-2’), 6.46 (dd, *J* = 7.9, 1.5 Hz, 1H, H-6’), 4.10 (t, *J* = 6.8 Hz, 2H, CH_2_C*H*_2_O), 2.68 (t, *J* = 6.8 Hz, 2H, C*H*_2_CH_2_O), 2.01-1.90 (m, 3H, C*H* _adamantyl_), 1.81-1.73 (m, 6H, C*H*_2 adamantyl_), 171-1.60 (m, 6H, C*H*_2 adamantyl_). ^13^C NMR (50 MHz, DMSO-*d6*) δ (ppm): 176.83 (*C*O), 145.48 (C-3’), 143.92 (C-4’), 129.12 (C-1’), 119.98 (C-6’), 116.72 (C-2’), 115.88 (C-5’), 64.83 (CH_2_*C*H_2_O), 40.00 (C _adamantyl_), 38.82 (*C*H_2 adamantyl_), 36.39 (*C*H_2 adamantyl_), 34.32 (*C*H_2_CH_2_O), 27.76 (*C*H _adamantyl_). HR-MS (ESI) m/z: Calcd for C_19_H_23_O_4_: [M1 - H]^-^ = 315.1601, found 315.1599.

3,4-dihydroxyphenethyl 3,5-dihydroxy benzoate (**14**)

The crude product was purified by column chromatography (silica gel) using a mixture of cyclohexane / ethyl acetate (1/1) as the eluent, affording the corresponding ester **14** in 84% yield.

Mp: 110-111 °C (EtOAc- c-Hex). ^1^H NMR (600 MHz, DMSO-*d6*) δ (ppm): 9.64 (br s, 2H, D_2_O exchang, 3-O*H* ,5-O*H*), 8.79 (br s, 1H, D_2_O exchang, 4’-O*H*), 8.75 (br s, 1H, D_2_O exchang, 3’-O*H*), 6.80 (d, *J* = 2.1 Hz, 2H, H-2, H-6), 6.67 (d, *J* = 7.9 Hz, 1H, H-5’), 6.64 (d, *J* = 2.1 Hz, 1H, H-2’), 6.53 (dd, *J* = 7.9, Hz, 2.1 Hz, 1H, H-6’), 6.44 (t, *J* = 2.3 Hz, 1H, H-4), 4.32 (t, *J* = 6.8 Hz, 2H, CH_2_C*H*_2_O), 2.81 (t, *J* = 6.8 Hz, 2H, C*H*_2_CH_2_O). ^13^C NMR (151 MHz, DMSO-*d6*) δ (ppm): 166.28 (*C*O), 158.82 (C-3, C-5), 145.65 (C-3’), 144.32 (C-4’), 132.03 (C-1), 129.25 (C-1’), 119.84 (C-6’), 116.78 (C-2’), 116.11 (C-5’), 107.55 (C-2, C-4, C-6), 66.04 (CH_2_*C*H_2_O), 34.37 (*C*H_2_CH_2_O). HR-MS (ESI) m/z: Calcd for C_15_H_13_O_6_: [M1 - H]^-^ = 289.0717, found 289.0713.

3,4-dihydroxyphenethyl octanoate (**15**)

The crude product was purified by column chromatography (silica gel) using a mixture of cyclohexane / ethyl acetate (4/1) as the eluent, affording the corresponding ester **15** in 91% yield, as an oil.

^1^H NMR (400 MHz, CDCl_3_) δ (ppm): 6.81 (d, *J* = 8.0 Hz, 1H, H-5’), 6.75 (d, *J* = 1.6 Hz, 1H, H-2’), 6.62 (dd, *J* = 8.0, 1.6 Hz, 1H, H-6’), 4.26 (t, *J* = 7.2 Hz, 2H, CH_2_C*H*_2_O), 2.82 (t, *J* = 7.2 Hz, 2H, C*H*_2_CH_2_O), 2.32 (t, *J* = 7.2 Hz, 2H, 2-C*H*_2_), 1.66–1.57 (m, 2H, 3-C*H*_2_), 1.22-1.36 (m, 8H, 4-C*H*_2_, 5-C*H*_2_, 6-C*H*_2_, 7-C*H*_2_), 0.90 (t, *J* = 7.2 Hz, 3H, C*H*_3_). ^13^C NMR (151 MHz, CDCl_3_) δ (ppm): 175.01 (*C*O), 143.80 (C-3’), 142.50 (C-4’), 130.31 (C-1’), 121.19 (C-6’), 115.86 (C-2’), 115.36 (C-5’), 65.28 (CH_2_*C*H_2_O), 34.47 (*C*H_2_CH_2_O), 34.42 (C-2), 31.63 (C-6), 29.05 (C-5), 28.89 (C-4), 24.94 (C-3), 22.59 (C-7), 14.05 (C-8). HR-MS (ESI) m/z: Calcd for C_16_H_23_O_4_: [M1 - H]^-^ = 279.1601, found 279.1590.

3,4-dihydroxyphenethyl 2-(adamantan-1-yl)acetate (**16**)

The crude product was purified by column chromatography (silica gel) using a mixture of cyclohexane / ethyl acetate (4/1) as the eluent, affording the corresponding ester **16** in 95% yield.

^1^H NMR (600 MHz, CDCl_3_) δ (ppm): 6.79 (d, *J* = 8.1 Hz, 1H, H-5’), 6.75 (d, *J* = 2.0 Hz, 1H, H-2’), 6.63 (dd, *J* = 8.1, 2.0 Hz, 1H, H-6’), 4.24 (t, *J* = 7.1 Hz, 2H, CH_2_C*H*_2_O), 2.82 (t, *J* = 7.1 Hz, 2H, C*H*_2_CH_2_O), 2.06 (s, 2H, C*H_2_*CO), 1.93 (m, 3H, C*H* _adamantyl_), 1.70–1.57 (m, 6H, C*H*_2 adamantyl_), 1.54 (m, 6H, C*H*_2 adamantyl_). ^13^C NMR (151 MHz, CDCl3) δ (ppm): 172.66 (*C*O), 143.72 (C-3’), 142.41 (C-4’), 130.51 (C-1’), 121.25 (C-6’), 115.85 (C-2’), 115.30 (C-5’), 64.88 (CH_2_*C*H_2_O), 49.14 (*C*H_2_CO), 42.39 (*C*H_2 adamantyl_), 36.68 (*C*H_2 adamantyl_), 34.47 (*C*H_2_CH_2_O), 32.82 (C _adamantyl_), 28.61 (*C*H _adamantyl_). HR-MS (ESI) m/z: Calcd for C_20_H_25_O_4_: [M1 - H]^-^ = 329.1758, found 329.1749.

(Z)-3,4-dihydroxyphenethyl docos-13-enoate (**17**)

The crude product was purified by column chromatography (silica gel) using a mixture of cyclohexane / ethyl acetate (4/1) as the eluent, affording the corresponding ester **17** in 95% yield.

^1^H NMR (600 MHz, CDCl_3_) δ (ppm): 6.78 (d, *J* = 8.0 Hz, 1H, H-5’), 6.73 (d, *J* = 2.0 Hz, 1H, H-2’), 6.63 (dd, *J* = 8.0, 2.0 Hz, 1H, H-6’), 5.35 (m,2H, C*H*=C*H*), 4.24 (t, *J* = 7.1 Hz, 2H, CH_2_C*H*_2_O), 2.81 (t, *J* = 7.1 Hz, 2H, C*H*_2_CH_2_O), 2.29 (t, *J* = 7.6 Hz, 2H, COC*H*_2_), 2.01 (m, 4H, 12-C*H*_2,_ 15-C*H*_2_), 1.61 (m, 2H, 3-C*H*_2_), 1.38–1.17 (m, 28H, C*H*_2 erucic_), 0.88 (t, J = 6.9 Hz, 3H, C*H*_3_). ^13^C NMR (151 MHz, CDCl3) δ (ppm): 174.58 (*C*O), 143.81 (C-3’), 142.46 (C-4’), 130.74 (C-1’), 130.06 (*C*H=*C*H), 121.42 (C-6’), 116.02 (C-2’), 115.49 (C-5’), 65.18 (CH_2_*C*H_2_O), 34.58 (*C*H_2_CH_2_O), 33.82 (C-2), 32.00 (C-20), 30.24–28.81 (*C*H_2 erucic_), 27.37 (C-12, C-15), 25.02 (C-3), 22.81 (C-21), 14.24 (*C*H_3_). HR-MS (ESI) m/z: Calcd for C_30_H_49_O_4_: [M1 - H]^-^ = 473.3636, found 473.3636.

3,4-dihydroxyphenethyl 4-isopropylcyclohexanecarboxylate (**18**)

The crude product was purified by column chromatography (silica gel) using a mixture of CH_2_Cl_2_ / MeOH (100/1) as the eluent, affording the corresponding ester **18** in 96% yield.

^1^H NMR (600 MHz, CDCl_3_) δ (ppm): 6.78 (d, *J* = 8.1 Hz, 1H, H-5’), 6.73 (d, *J* = 2.0 Hz, 1H, H-2’), 6.63 (dd, *J* = 8.1, 2.0 Hz, 1H, H-6’), 5.60 (br s, 1H, D_2_O exchang., 4’-O*H*), 5.40 (br s, 1H, D_2_O exchang., 3’-O*H*), 4.22 (t, *J* = 7.1 Hz, 2H, CH_2_C*H*_2_O), 2.81 (t, *J* = 7.1 Hz, 2H, C*H*_2_CH_2_O), 2.25 – 2.14 (m, 1H, C*H*), 1.98 – 1.93 (m, 2H, C*H*), 1.80 – 1.73 (m, 2H, C*H*), 1.44 – 1.32 (m, 3H, C*H*), 1.06 – 0.93 (m, 3H, C*H*), 0.85 (d, *J* = 6.8 Hz, 6H, C*H*_3_). ^13^C NMR (151 MHz, CDCl_3_) δ (ppm): 176.82 (*C*O), 143.77 (C-3’), 142.39 (C-4’), 130.90 (C-1’), 121.47 (C-6’), 116.05 (C-2’), 115.46 (C-5’), 65.02 (CH2*C*H2O), 43.80 (*C*H), 43.42 (*C*H), 34.64 (*C*H_2_CH_2_O), 32.91 (*C*H), 29.35 (*C*H_2_), 29.01 (*C*H_2_), 19.88 (*C*H_3_). HR-MS (ESI) m/z: Calcd for C_18_H_25_O_4_: [M1 - H]^-^ = 305.1758, found 305.1749.

3,4-dihydroxyphenethyl 3,5,5-trimethylhexanoate (**19**)

The crude product was purified by column chromatography (silica gel) using a mixture of cyclohexane / CH_2_Cl_2_ (1/2) as the eluent, affording the corresponding ester **19** in 95% yield.

^1^H NMR (600 MHz, CDCl_3_) δ (ppm): 6.78 (d, *J* = 8.0 Hz, 1H, H-5’), 6.73 (d, *J* = 2.0 Hz, 1H, H-2’), 6.61 (dd, *J* = 8.0, 2.0 Hz, 1H, H-6’), 6.22 (br s, 1H, D_2_O exchang., 4’-O*H*), 6.13 (br s, 1H, D_2_O exchang., 3’-O*H*), 4.22 (t, *J* = 7.1 Hz, 2H, CH_2_C*H*_2_O), 2.80 (t, *J* = 7.1 Hz, 2H, C*H*_2_CH_2_O ), 2.29 (m, 1H, C*H*), 2.10 (m, 1H, C*H*), 2.03 – 1.95 (m, 1H, C*H*), 1.20 (m, 1H, C*H*), 1.09 (m, 1H, C*H*), 0.93 (d, *J* = 6.6 Hz, 3H, CH_3_), 0.88 (s, 9H, C*H*_3_). ^13^C NMR (151 MHz, CDCl_3_) δ (ppm): 173.52 (*C*O), 143.78 (C-3’), 142.37 (C-4’), 130.95 (C-1’), 121.47 (C-6’), 116.08 (C-2’), 115.50 (C-5’), 64.99 (CH_2_*C*H_2_O), 50.68 (*C*H_2_CH), 44.21 (*C*H_2_CH), 34.65 (*C*H_2_CH_2_O), 31.19 (C), 30.07 (3 x C*H*_3_), 27.17 (*C*H), 22.78 (C*H*_3_). HR-MS (ESI) m/z: Calcd for C_17_H_25_O_4_: [M1 - H]^-^ = 293.1758, found 293.1750.

3,4-dihydroxyphenethyl 2-cyclohexylacetate (**20**)

The crude product was purified by column chromatography (silica gel) using a mixture of cyclohexane / ethyl acetate (4/1) as the eluent, affording the corresponding ester **20** in 95% yield, as an oil.

^1^H NMR (400 MHz, CDCl_3_) δ (ppm): 6.81 (d, *J* = 8.0 Hz, 1H, H-5’), 6.75 (d, *J* = 1.8 Hz, 1H, H-2’), 6.62 (dd, *J* = 8.0, 1.8 Hz, 1H, H-6’), 4.27 (t, *J* = 7.0 Hz, 2H, CH_2_C*H*_2_O), 2.84 (t, *J* = 7.0 Hz, 2H, C*H*_2_CH_2_O), 2.19 (d, *J* = 7.1 Hz, 2H, COC*H*_2_), 1.82–1.60 (m, 6H, H-1, H-2, H-3, H-4, H-5, H-6), 1.31–1.08 (m, 3H, H-3, H-4, H-5), 0.98–0.91 (m, 2H, H-2, H-6). ^13^C NMR (151 MHz, CDCl_3_) δ (ppm): 174.33 (*C*O), 143.89 (C-3’), 142.58 (C-4’), 130.18 (C-1’), 121.07 (C-6’), 115.83 (C-2’), 115.31 (C-5’), 65.23(CH_2_*C*H_2_O), 42.29 (CO*C*H_2_), 34.89 (*C*H_2_CH_2_O), 34.41 (C-1), 32.94 (C-2, C-6), 26.90 (C-4), 26.06 (C-3, C-5). HR-MS (ESI) m/z: Calcd for C_16_H_21_O_4_: [M1 - H]^-^ = 277.1445, found 277.1436.

**General Procedure for the synthesis of compounds 21-24**

A solution of the appropriate ester (**2**, **3**, **4** or **11**) (1 mmol) in t-butanol (20 ml) was hydrogenated in the presence of 10% Pd/C (50 mg), under a pressure of 50 psi at room temperature for 3-4 h. After completion of the reaction, the resulting mixture was filtered through a celite pad and the filtrate was evaporated to dryness to afford the title compounds **21**-**24**.

2-(3,4-dihydroxyphenyl)-2-hydroxyethyl adamantane-1-carboxylate (**21**)

The crude product was purified by column chromatography (silica gel) using a mixture of cyclohexane / ethyl acetate (2/1) as the eluent, affording the corresponding ester **21** in 81% yield.

Mp: 148-149 °C (CHCl_3_/*n*-pentane). ^1^H NMR (400 MHz, DMSO-*d6*) δ (ppm): 8.80 (br s, 1H, D_2_O exchang., 3’-O*H*), 8.73 (br s, 1H, D_2_O exchang., 4’-O*H*), 6.76 (d, *J* = 1.9 Hz, 1H, H-2’), 6.67 (d, *J* = 8.0 Hz, 1H, Η-5’), 6.59 (dd, *J* = 8.0, 1.9 Hz, 1H, Η-6’), 5.30 (d, *J* = 4.4 Hz, 1H, CHO*H*), 4.58-4.53 (m, 1H, C*H*CH_2_), 4.00–3.90 (m, 2H, CHC*H*_2_), 1.98-1.94 (m, 3H, C*H* _adamantyl_), 1.81-1.75 (m, 6H, C*H*_2 adamantyl_), 1.71-1.61 (m, 6H, C*H*_2 adamantyl_). ^13^C NMR (151 MHz, DMSO-*d6*) δ (ppm): 176.32 (*C*O), 144.87 (C-3’), 144.42 (C-4’), 133.14 (C-1’), 117.15 (C-6’), 115.02 (C-2’), 113.74 (C-5’), 70.08 (HO*C*HCH_2_), 68.41 (HOCH*C*H_2_), 40.05 (C _adamantyl_), 38.30 (*C*H_2 adamantyl_), 35.93 (*C*H_2 adamantyl_), 27.28 (*C*H _adamantyl_). HR-MS (ESI) m/z: Calcd for C_19_H_23_O_5_: [M1 - H]^-^ = 331.1551, found 331.1545.

2-(3,4-dihydroxyphenyl)-2-hydroxyethyl 3,5-dihydroxybenzoate (**22**)

The crude product was purified by column chromatography (silica gel) using a mixture of cyclohexane / ethyl acetate (1/1) as the eluent, affording the corresponding ester **22** in 76% yield.

Mp: 181-182 °C (CH_2_Cl_2_). ^1^H NMR (400 MHz, DMSO-*d6*) δ (ppm): 9.64 (br s, 2H, D_2_O exchang., 3-O*H*, 5-O*H*), 8.87 (br s, 1H, D_2_O exchang., 3’-O*H*), 8.82 (br s, 1H, D_2_O exchang., 4’-O*H*), 6.83 (s, 2H, H-2, H-6), 6.81 (s, 1H, H-2’), 6.69 (d, *J* = 8.0 Hz, 1H, Η-5’), 6.65 (d, *J* = 8.0 Hz, 1H, Η-6’), 6.44 (s, 1H, H-4), 5.47 (m, 1H, CHO*H*), 4.75-4.65 (m, 1H, C*H*CH_2_), 4.20-4.10 (m, 2H, CHC*H*_2_). ^13^C NMR (151 MHz, DMSO-*d6*) δ (ppm): 166.00 (*C*O), 158.46 (C-3,5), 145.00 (C-3’), 144.67 (C-4’), 132.82 (C-1’), 131.30 (C-1), 116.99 (C-6’), 115.04 (C-2’), 113.41 (C-5’), 107.26 (C-2, C-4, C-6), 69.93 (*C*HCH_2_), 69.23 (CH*C*H_2_). HR-MS (ESI) m/z: Calcd for C_15_H_13_O_7_: [M1 - H]^-^ = 305.0667, found 305.0654.

2-(3,4-dihydroxyphenyl)-2-hydroxyethyl octanoate (**23**)

The crude product was purified by column chromatography (silica gel) using a mixture of cyclohexane / ethyl acetate (2/1) as the eluent, affording the corresponding ester **23** in 87% yield.

Mp: 119-120 °C (CH_2_Cl_2_/*n*-pentane). ^1^H NMR (400 MHz, DMSO-*d6*) δ (ppm): 8.84 (br s, 1H, D_2_O exchang., 3’-O*H*), 8.77 (br s, 1H, D_2_O exchang., 4’-O*H*), 6.75 (d, *J* = 2.0 Hz, 1H, H-2’), 6.66 (d, *J* = 8.0 Hz, 1H, Η-5’), 6.57 (dd, *J* = 8.0 Hz, 2.0 Hz, 1H, Η-6’), 5.34 (m, 1H, CHO*H*), 4.61-4.52 (m, 1H, C*H*CH_2_), 4.00–3.90 (m, 2H, CHC*H*_2_), 2.26 (t, *J* = 7.3 Hz, 2H, 2-C*H*_2_), 1.56-1.44 (m, 2H, 3-C*H*_2_), 1.34-1.14 (m, 8H, 4-C*H*_2_, 5-C*H*_2_, 6-C*H*_2_, 7-C*H*_2_), 0.85 (t, *J* = 7.3 Hz, 3H, C*H*_3_). ^13^C NMR (151 MHz, DMSO-*d6*) δ (ppm): 172.82 (*C*O), 144.93 (C-3’), 144.51 (C-4’), 132.97 (C-1’), 117.09 (C-6’), 115.10 (C-2’), 113.68 (C-5’), 69.83 (HO*C*HCH_2_), 68.79 (HOCH*C*H_2_), 33.37 (C-2), 31.09 (C-6), 28.36 (C-4, C-5), 24.41 (C-3), 22.03 (C-7), 13.93 (C-8). HR-MS (ESI) m/z: Calcd for C_16_H_23_O_5_: [M1 - H]^-^ = 295.1550, found 295.1543.

2-(3,4-dihydroxyphenyl)-2-hydroxyethyl 3,5,5-trimethylhexanoate (**24**)

The crude product was purified by column chromatography (silica gel) using a mixture of CH_2_Cl_2_ / MeOH (100/1) as the eluent, affording the corresponding ester **24** in 94% yield, as an oil.

^1^H NMR (600 MHz, Acetone-*d6*) δ (ppm): 7.08 (br s, 1H, D_2_O exchang., O*H*), 6.92 (d, *J* = 1.9 Hz, 1H, H-2’), 6.78 (d, *J* = 8.0 Hz, 1H, Η-5’), 6.74 (dd, *J* = 8.0, 1.9 Hz, 1H, Η-6’), 4.76 (m, 1H, CHO*H*), 4.15-4.03 (m, 2H, C*H*CH_2_), 2.29 (m, 1H, C*H*), 2.15-2.07 (m, 2H, 2 x C*H*), 1.28 (m, 1H, C*H*), 1.10 (m, 1H, C*H*), 0.95 (d, *J* = 6.6 Hz, 3H, CH_3_), 0.91 (s, 9H, C*H*_3_). ^13^C NMR (151 MHz, Acetone-*d6*) δ (ppm): 172.11 (*C*O), 144.85 (C-3’), 144.47 (C-4’), 133.64 (C-1’), 117.75 (C-6’), 114.85 (C-2’), 113.39 (C-5’), 71.12 (HO*C*HCH_2_), 68.91 (HOCH*C*H_2_), 50.27 (*C*H_2_CH), 43.47 (*C*H_2_CH), 30.62 (C), 29.34 (3 x C*H*_3_), 26.75 (*C*H), 22.07 (C*H*_3_). HR-MS (ESI) m/z: Calcd for C_17_H_25_O_5_: [M1 - H]^-^ = 309.1707, found 309.1696.

**

Supplementary figures**

**Supplementary Figure 1**. Synthesized analogs of HT

**
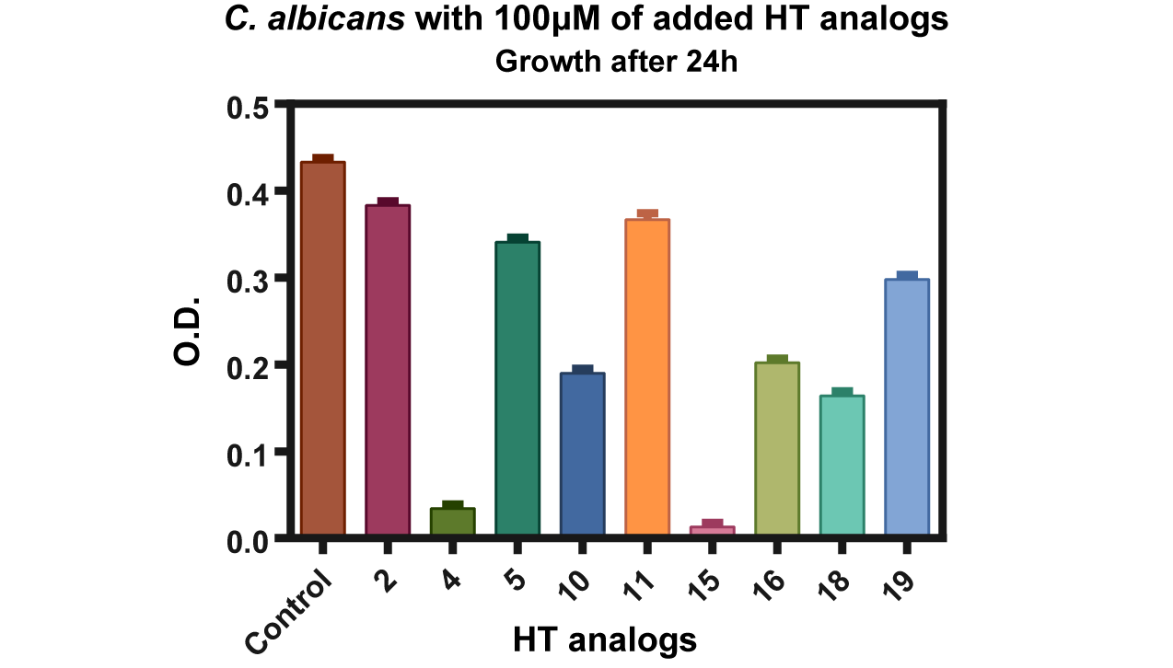
**

**Supplementary Figure 2.** Overnight growth of *C. albicans* in the presence of HT analogs. Control stands for a culture where instead of an HT analog, DMSO solvent was added in a concentration identical to that in which HT analogs were dissolved. Growth is recorded after 24 h as O.D. values at 600 nm.

**
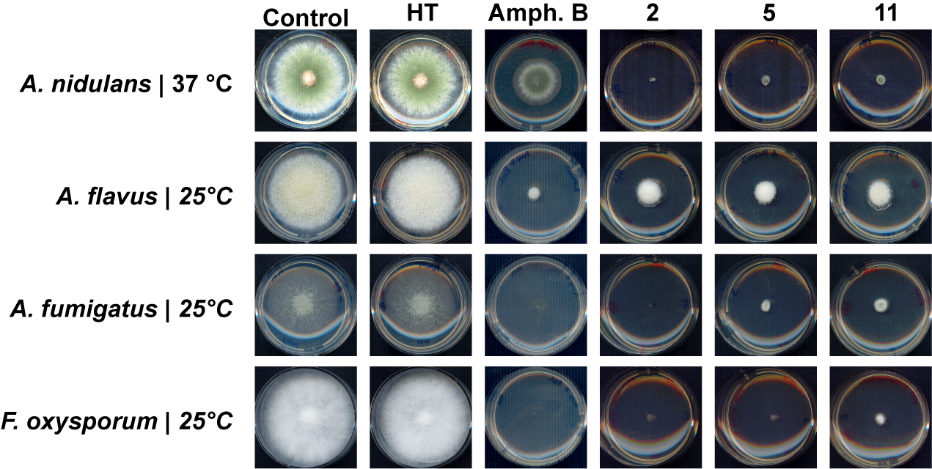
**

**Supplementary Figure 3.** Comparison of antifungal activity of HT, amphotericin B and selected HT analogs. The concentration of antifungals shown is 100 μΜ. Control stands for a culture where DMSO solvent was added in a concentration identical to that of antifungals.

**
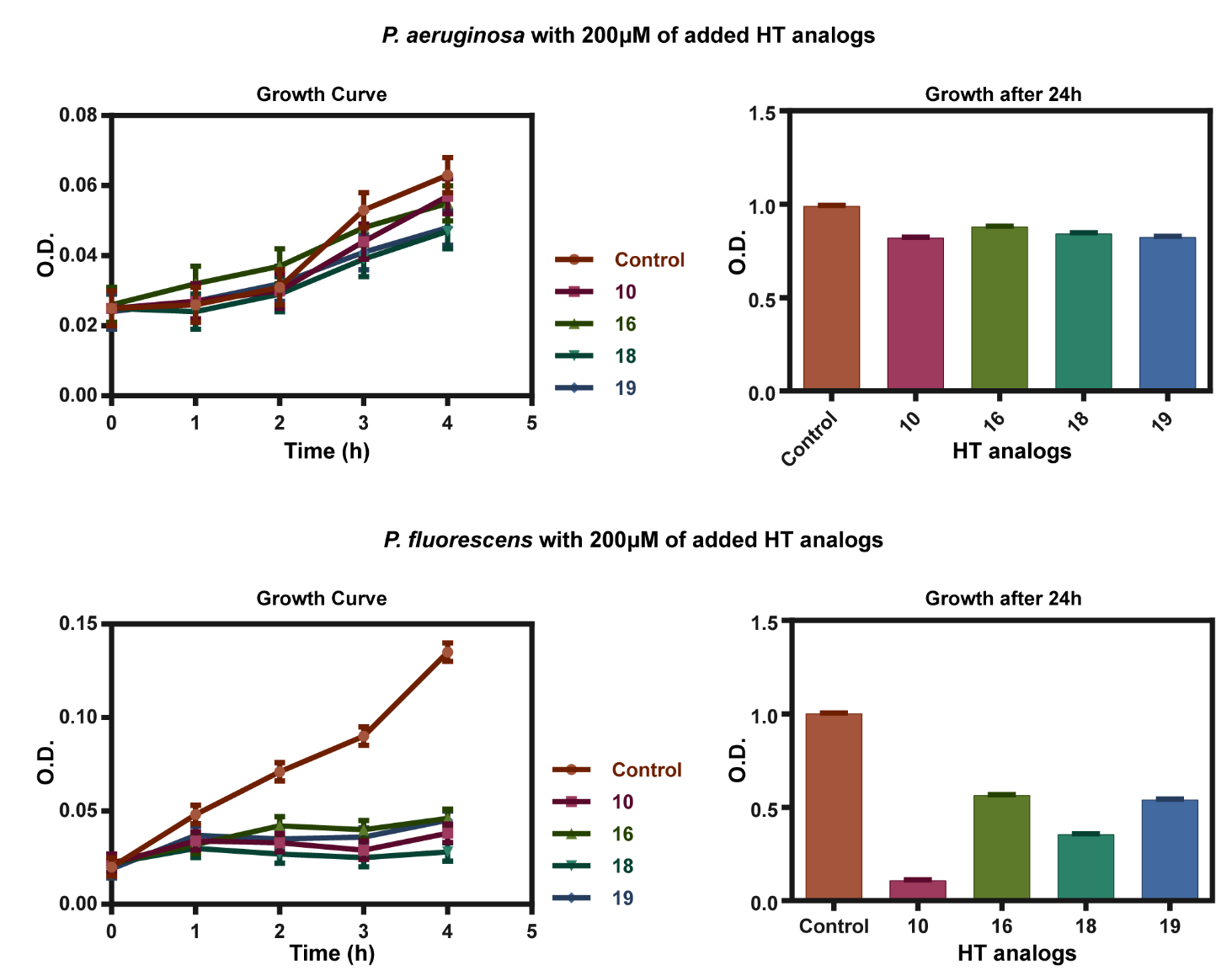
Supplementary Figure 4. Antibacterial activity of HT analogs against *P. aeruginosa* and *P. fluorescens.*** Growth curves on the left show O.D. values recorded hourly at 600 nm. Column bar graphs on the right show the growth after 24 hours after the HT analogs addition, at 600 nm. Control stands for a culture DMSO solvent was added in a concentration identical to that of HT analogs.

*
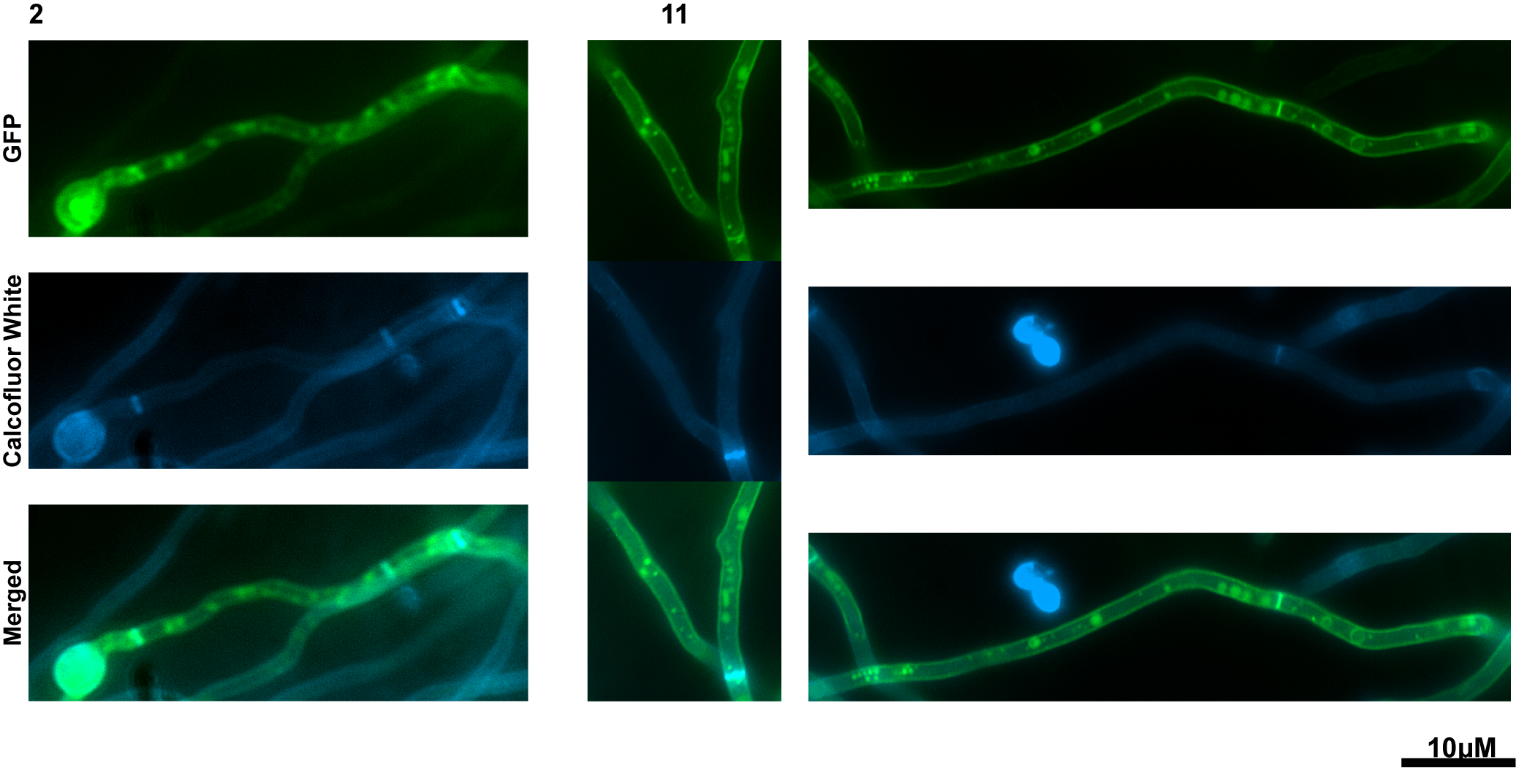
*

**Supplementary Figure 5. Epifluorescence *in vivo* microscopy showing the apparent non-effect of HT analogs 2 and 11 (37.5 μΜ) on *A. nidulans* cell wall**. The picture shows hyphae of strains expressing functional, GFP-tagged, FurA as PM molecular marker, stained with Calcuofluor white as a standard marker for cell wall integrity (Martzoukou et al., 2017).
